# Muscle strength, an independent determinant of glycemic control in older adults with long-standing type 2 diabetes: a prospective cohort study

**DOI:** 10.1186/s12877-021-02629-5

**Published:** 2021-12-07

**Authors:** Bo Kyung Koo, Seoil Moon, Min Kyong Moon

**Affiliations:** 1 Department of Internal Medicine, Seoul National University College of Medicine, Seoul, Republic of Korea; 2 Division of Endocrinology, Department of Internal Medicine, Seoul National University Boramae Medical Center, Seoul, Republic of Korea; 3 Department of Internal Medicine, Seoul National University Hospital, Seoul, Republic of Korea

**Keywords:** Diabetes mellitus, type 2, Older adult, Sarcopenia, Handgrip strength

## Abstract

**Background:**

Although the proportion of older patients with type 2 diabetes mellitus (T2DM) has increased, few studies have reported the factors affecting glucose levels in older patients with long-standing T2DM. This study assessed the determinants of glycemic control in older adults with T2DM of a duration of ≥10 years, including muscle mass, muscle quality, and β-cell function.

**Methods:**

This was a prospective study of older patients aged ≥60 years with a T2DM duration of ≥10 years. The Homeostatic Model Assessment of Insulin Resistance (HOMA-IR) index, handgrip strength (HGS), and body composition through bioelectrical impedance analysis were assessed. The primary outcome was a composite of: (i) increment of glycated hemoglobin (HbA1c) from the baseline ≥0.6% and (ii) HbA1c ≥ 9% at any time point during the follow-up period. To find the predicting determinants of the outcome, we performed the Cox proportional hazard analysis.

**Results:**

Among 100 patients (mean age, 64.0 ± 8.6 years; median duration of diabetes, 20 [interquartile range (IQR), 17–23] years; median HbA1c at baseline, 7.1 [IQR, 6.7–7.4] %), the primary outcome was observed in 40 (40.0%) patients during 4.0 (IQR 2.3–5.0) years of follow-up. A Cox proportional hazards model adjusted for age, sex, baseline HbA1c, obesity, duration of DM and anti-diabetic medication at baseline showed that low HGS and insulin resistance at the baseline were independent determinants of the primary outcome (hazard ratio [HR] = 2.23 [95% confidence interval (CI), 1.06–4.72] and 2.39 [95% CI, 1.18–4.83], respectively). Sex stratification confirmed that HGS and muscle mass were independent determinants of the primary outcome only in women (HR per quartile, 0.58 [95% CI, 0.37–0.93] and 0.46 [95% CI, 0.25–0.85], respectively). `.

**Conclusions:**

Low HGS and insulin resistance were independent risk factors for aggravated glycemic control among older patients with long standing T2DM.

**Supplementary Information:**

The online version contains supplementary material available at 10.1186/s12877-021-02629-5.

## Background

The rate of failure of anti-diabetic agents in patients with long-standing diabetes mellitus (DM) is high; this is because of the associated progressive β-cell dysfunction in type 2 diabetes mellitus (T2DM) [1–3]. A previous study reported that among patients with a T2DM duration ≥10 years, the proportion of patients who were on insulin were above 25% of the study population, and this proportion increased to about 50% among patients with a T2DM duration of 20 years [2]. With the increasing life expectancy, the proportion of older patients with diabetes has increased [4, 5]. Most of these patients have long-standing DM, which results in a high prevalence of diabetic complications and increased mortality risk [6]. Early aggressive glycemic controls [7], medications [3, 7], physical activity [8], and quality of self-care are determinants of glycemic control in T2DM; however, few studies have reported the factors affecting glucose levels in older patients with long-standing DM.

Older patients with DM tend to have coexisting cognitive dysfunction [9], functional disability, emotional lability [10], and other comorbidities, such as cardiovascular diseases (CVD), stroke, or malignancies; these co-existing diseases are more frequent in young patients with DM [6] and are barriers to proper glycemic control [9, 11]. In addition, economic dependency of the older population makes their management difficult [12]. In Korea, despite the high prevalence of DM in the population ≥ 65 years old, only about 30% of them reach the target HbA1c [5].

Aging reduces normal β-cell function [11], and β-cell senescence is more prominent in patients with DM compared to non-diabetic individuals [13]. In addition, sarcopenia and increasing fat mass that occur during aging are factors that aggravate glycemic control in older patients [14]. There are few studies elucidating the role of residual β-cell function and sarcopenia on glycemic control in older patients with long-standing DM. A recent cross-sectional study with older patients with DM showed that poor glycemic control was associated with low muscle mass [15]. Therefore, this study sought to identify the determinants of glycemic control in older patients with T2DM of a disease duration ≥10 years, including muscle mass, muscle quality, and β-cell function.

## Methods

### Study subjects

This was a prospective cohort study. Patients who were at least 60 years old, diagnosed with T2DM at least 10 years ago, and had results of two consecutive oral glucose tolerance tests (OGTTs) performed 3 years apart were eligible for this study. The exclusion criteria were: (1) patients with baseline HbA1c ≥ 9%, (2) patients receiving insulin for glycemic control, or (3) patients with a history of malignancy or systemic steroid therapy. No specific protocol for the prescription of anti-diabetic medication was used in this study. The routine clinical settings and guidelines were used in managing the patients with DM [16]. For the evaluation of insulin resistance and β-cell function, the participants underwent OGTT at baseline and 3 years thereafter. The protocol was approved by the Institutional Review Board of the Seoul National University Boramae Medical Center (IRB No. 06–2011–93). Written informed consent was obtained from all the participants.

### Study outcomes

The primary outcome was a composite of (i) increment of HbA1c from the baseline (ΔHbA1c) ≥ 0.6% and (ii) HbA1c ≥ 9% at any time point during the follow-up period irrespective of the baseline value. The secondary outcome was a composite of (a) the two primary outcomes and (b) change of medication due to hyperglycemia. For (b), the HbA1c level at the time of medication change was not considered to define an event. The patients were divided into two groups: the aggravation group with the primary outcome and the maintenance group without the primary outcome. Considering that the glycemic targets for older patients range from HbA1c 7.0% to HbA1c 8.5% depending on life expectancy, comorbidities, complications and physical status of the patients [17], HbA1c ≥ 9% was adopted to define aggravation. In addition, change of medication as an add-on therapy due to hyperglycemia would be resulted in 0.4 ~ 0.6% of HbA1c reduction [18–21], ΔHbA1c ≥ 0.6% was added to the primary outcome.

### Metabolic parameters measurement

During the 75-g OGTT, the plasma glucose and insulin level at 0 and 30 min were measured to assess insulin resistance and insulin secretion capacity. The insulinogenic index (IGI) was calculated using the following formula from OGTT as an index of β-cell function: [Δinsulin (uU/mL) _0–30 min_/Δglucose (mg/dL) _0–30 min_] [22]. The Homeostatic Model Assessment for β-cell (HOMA-β) index was calculated using [20 × fasting glucose (mmol/L)]/[fasting glucose (mmol/L) – 3.5] [22]. The Homeostatic Model Assessment for Insulin Resistance (HOMA-IR) index was calculated using: [fasting glucose (mmol/L) × fasting insulin (uU/mL)]/22.5 [22]; insulin resistance was defined as a HOMA-IR index ≥2.5 [23].

Hypertension (HTN) was defined as a systolic blood pressure (BP) ≥ 140 mmHg, diastolic BP ≥ 90 mmHg, or ongoing treatment with antihypertensive medications. Obesity and abdominal obesity were defined as a body mass index (BMI) ≥ 25 kg/m^2^ [24] and waist circumference ≥ 90 and ≥ 85 cm in men and women, respectively [25]. Elevated high-sensitivity C-reactive protein (hs-CRP) was defined as levels ≥1 mg/L [26]. Renal function was calculated using the modification of diet in renal disease (MDRD) estimated glomerular filtration rate (eGFR) [27].

### Assessment of muscle and fat mass and hand grip strength

Handgrip strength (HGS) was assessed using a digital grip strength dynamometer (TKK 5401; Takei, Tokyo, Japan), as previously reported [28]. Briefly, HGS was measured with the patient standing upright and elbow in full extension. The participants were instructed to squeeze the dynamometer as strongly as possible, for at least three seconds. Low HGS was defined as < 27 kg in men and < 16 kg in women, respectively, following the revised guidelines of the European Working Group on Sarcopenia in Older People [29]. To estimate the dose-response relationship between HGS and the risk of outcome, the quartiles of muscle strength were calculated for each sex, with Q_1_ and Q_4_ being the lowest and highest quartiles, respectively.

Bioelectrical impedance analysis was performed using the InBody 330 body composition analyzer (InBody, Seoul, Korea), which measures the muscle and fat mass. Muscle mass and fat mass were expressed as a percentage (muscle/weight [muscle%] or fat/weight [fat%]), and their quartiles were calculated for each sex.

### Statistical analysis

All data were analyzed using IBM SPSS Statistics 20.0 for Windows (IBM Inc., Chicago, IL, USA). The demographic and clinical data of the patients with and without glucose deterioration were compared using the Mann-Whitney test, an independent t-test, and a chi-square test. The Cox proportional hazards model was used to investigate the predicting factors for primary or secondary outcomes after adjustments for sex, age, baseline HbA1c, obesity, and duration of diabetes. The level of statistical significance was set at *P* < 0.05.

## Results

### Baseline characteristics

A total of 186 patients were eligible in the study. Among them, 100 patients underwent two consecutive OGTTs and were enrolled in this study (mean age 64.0 ± 8.6 years; men, 49.0%). Their duration of diabetes was 20 years (interquartile range [IQR], 17–23 years). The baseline median HbA1c and BMI were 7.1% (IQR, 6.7–7.4%) and 24.2 kg/m^2^ (IQR, 22.1–26.6 kg/m^2^), respectively. Metformin, dipeptidyl peptidase-IV (DPP4) inhibitors, and sulfonylurea (SU) were used in 91.0, 64.0, and 68.0% of the patients at baseline (Table 1).

The median follow-up duration was 4.0 (IQR 2.3–5.0) years. The primary and secondary outcomes were observed in 40 and 78 patients, respectively during the follow-up period. Each outcome was detected in 38 (38.0%), 14 (14.0%), and 71 (71.0%) patients for ΔHbA1c ≥ 0.6%, HbA1c ≥ 9%, and medication change, respectively. The median HbA1c at the development of each outcome was 7.85, 9.45, and 8.00%, respectively (Supplementary Table S1).

There was no difference in age, duration of diabetes, and baseline HbA1c between the aggravation and maintenance groups (Table 1). BMI, C-peptide, HOMA-IR index, IGI, HOMA β-cell index, low-density lipoprotein cholesterol, high-density lipoprotein cholesterol, triglyceride, aspartate aminotransferase, alanine aminotransferase, alanine aminotransferase, and GFR were not significantly different between the two groups (Table 1). Among the anti-diabetic medication, thiazolidinedione (TZD) was used more in the maintenance group than in the aggravation group (18.3% vs. 2.5%, *P* = 0.017; Table 1). SU and DPP4 inhibitors were used more in the aggravation group than in the maintenance group; the difference was not significant (*P* = 0.096 and 0.061, respectively; Table 1).

Comparing the subjects with and without secondary outcome, there were no differences in age, duration of diabetes, or baseline HbA1c between both groups (Supplementary Table S2). The HOMA-IR index was significantly higher in those with secondary outcome (2.56 [IQR, 2.02–4.01] vs. 2.12 [IQR, 1.66–3.04]; *P* = 0.038); however, there was no difference in IGI and HOMA β-cell index between the two groups (Supplementary Table S2). TZD was used less in the patients with secondary outcome than in those without (9.0% vs. 22.7%; *P* = 0.080; Supplementary Table S2).

### Determinants of the primary and secondary outcomes

**Fig. 1:**
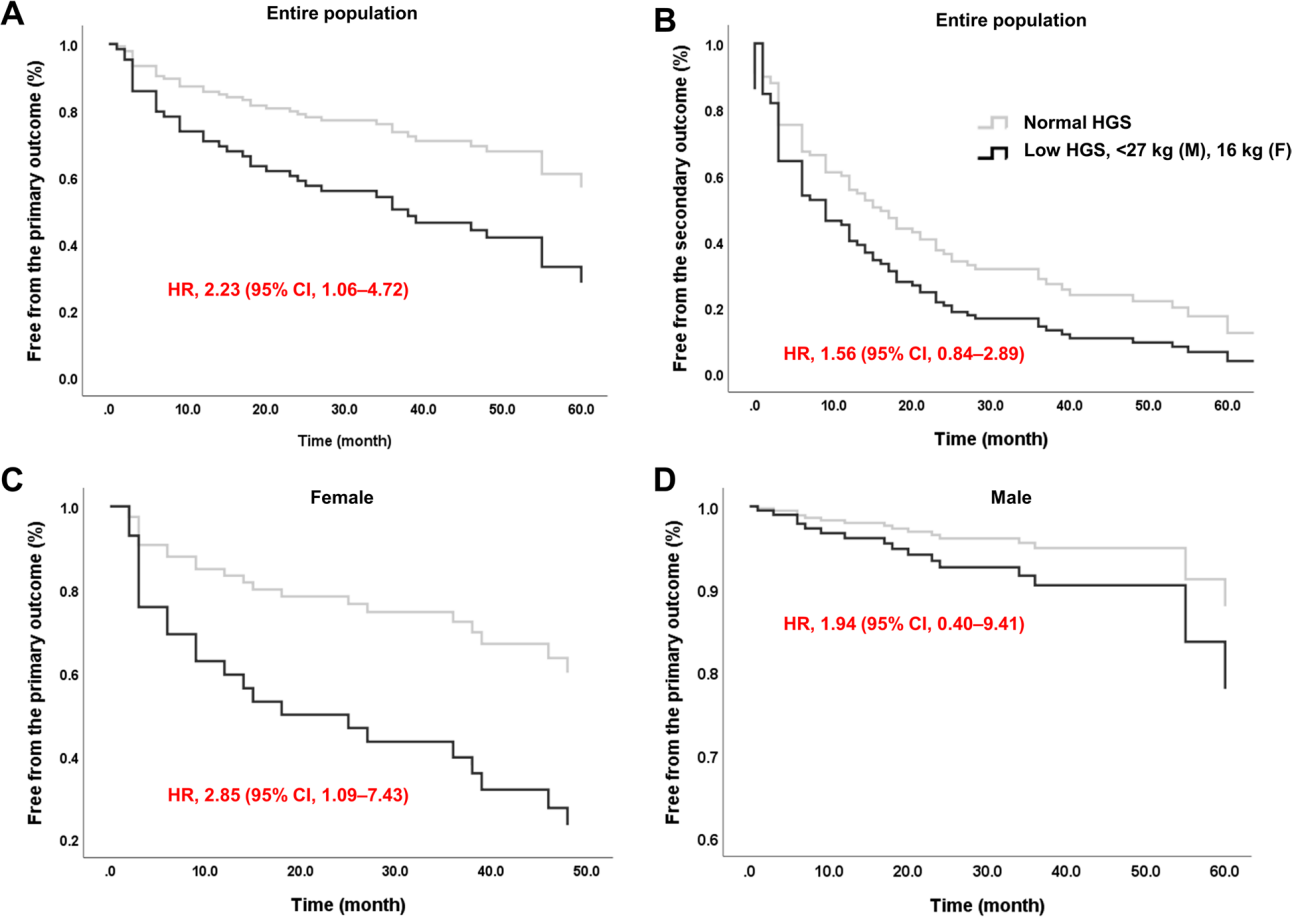
Risk of aggravated glycemic control according to handgrip strength (HGS). The Cox proportional hazards analysis after adjustment for age, sex, baseline HbA1c, obesity, duration of DM and anti-diabetic medication at baseline showed that low HGS, defined as < 27 kg (men), 16 kg (women), was independently associated with the primary outcome (**A**; HR, 2.234; 95% CI, 1.058–4.718) but not with the secondary outcome (**B**; HR, 1.556; 95% CI, 0.838–2.887) in the entire study population. Sex stratification confirmed the significant association between the primary outcome and HGS only in women (**C**; HR, 2.846; 95% CI, 1.090–7.434) and not in men (**D**; HR, 1.937; 95% CI, 0.399–9.410). DM, diabetes mellitus; HR, hazard ratio; CI, confidence interval

A Cox proportional hazards analysis was performed to investigate the determinants of the primary and secondary outcomes (Table 2). Among the various factors, low HGS increased the risk of the primary outcome (unadjusted hazard ratio [HR], 2.274; 95% confidence interval [CI], 1.132–4.567; *P* = 0.021) significantly, whose statistical significance was maintained even after adjustment for age, sex, baseline HbA1c, obesity, duration of DM, and anti-diabetic medication at baseline (HR, 2.234; 95% CI, 1.058–4.718 *P* = 0.035; Model 2 in Table 2; Fig. 1A). The baseline HOMA-IR index ≥2.5 also increased the risk of the primary outcome, which further increased after adjustment (Model 2; HR, 2.386; 95% CI, 1.178–4.834; *P* = 0.016). IGI, HOMA β-cell index, muscle mass/body weight (BW), fat mass/BW, and obesity at the baseline did not significantly affect the primary outcome (Table 2).

For the secondary outcome, only a HOMA-IR index ≥2.5 at the baseline increased the risk in the fully adjusted model (HR, 1.705; 95% CI, 1.048–2.775; *P* = 0.032). There was no significant association between low HGS and secondary outcome (Table 2, Fig. 1B).

Subsequently, the sex-stratified Cox proportional hazard model was analyzed. In women, there was association of low HGS and HOMA-IR index ≥2.5 with an increased risk of the primary outcome, even after adjustment for age, sex, baseline HbA1c, obesity, duration of DM and anti-diabetic medication at baseline (HR, 2.846; 95% CI, 1.090–7.434 and HR, 3.810; 95% CI, 1.307–11.100, respectively; Supplementary Table S3; Fig. 1C). As HGS increased by one quartile, the risk of the primary outcome decreased by 42% (HR, 0.584; 95% CI, 0.367–0.929; *P* = 0.023; Supplementary Fig. S1A). In addition, both muscle mass/BW and fat mass/BW were significant determinants of the primary outcome (Supplementary Table S3). Muscle mass/BW decreased the risk by 54% in every quartile (HR, 0.458; 95% CI, 0.249–0.850). In contrast, fat mass/BW increased the risk of primary outcome by about two times in every quartile (HR, 1.970; 95% CI, 1.058–3.667; Supplementary Table S3). The secondary outcome was also significantly associated with a low HGS and HOMA-IR index ≥2.5 in women (HR, 2.298; 95% CI, 1.002–5.374 and HR, 2.264; 95% CI, 1.044–4.910, respectively).

In men, low HGS and HOMA-IR index ≥2.5 increased the risk of the primary outcome (HR, 1.937; 95% CI, 0.399–9.410 and HR 2.155; 95% CI, 0.748–6.205, respectively; Fig. 1D); however, none of them were significant (Supplementary Table S3). A dose-response relationship between HGS and the risk of the primary outcome was not found in men (Supplementary Fig. S1B).

Low HGS was the most important factor for the primary and secondary outcomes. An additional analysis was conducted with each composite outcome for aggravation of glucose levels according to low HGS. Low HGS significantly increased the risk of two composite outcomes after adjustment for age, sex, and baseline HbA1c for ΔHbA1c ≥ 0.6% (HR, 2.793; 95% CI, 1.334–5.847; *P* = 0.006) and for HbA1c ≥ 9% (HR, 3.313; 95% CI, 1.059–10.361; *P* = 0.040), but not medication change (HR, 1.707; 95% CI, 0.912–3.193; *P* = 0.094). Even after additional adjustment for obesity, duration of diabetes and anti-diabetic medication at baseline, low HGS significantly increased the risk of ΔHbA1c ≥ 0.6% (HR, 2.467; 95% CI, 1.149–5.300; *P* = 0.021; Supplementary Table S4).

## Discussion

This study identified the determinants of glycemic control in older patients with T2DM of a duration ≥10 years. Low HGS and insulin resistance were independent risk factors for aggravated glycemic control. Low HGS was associated with a 2.5 times higher risk of the primary outcome in women, which was more prominent compared to that in men. In addition, in women, the risk of aggravated glycemic control had a significant negative and positive correlation with fat mass and muscle mass, respectively. The surrogate markers for β-cell function did not affect glycemic control, regardless of sex.

Sarcopenia is a progressive and generalized skeletal muscle disorder characterized by low muscle strength, low muscle quantity/quality, and low physical performance [29]. Aging reduces all these sarcopenic indices, and T2DM worsens sarcopenia in older patients [30]. Previous cross-sectional studies showed that poor glycemic control was associated with poor muscle quality [31] and low muscle mass [15] in older patients with T2DM. This prospective study is in line with their findings as sarcopenia was an independent risk factor for aggravated glycemic control [15, 31].

HGS is a method for measuring muscle strength when diagnosing sarcopenia [32]. HGS is a risk indicator for T2DM in the general population [33]. A prospective cohort study performed over 10 years ago confirmed that a greater HGS prevents T2DM development in persons without diabetes [34]. This study adds evidence of the role of muscle quality on the deterioration of glucose metabolism among older patients with long-standing T2DM. Resistance training significantly improves glycemic control and muscle strength in older patients with T2DM [35]. Improving muscle quality and mass reduces weight gain, abdominal adiposity, insulin resistance, and chronic inflammation, which in turn reduces insulin resistance [36].

Interestingly, the effect of muscle mass and/or muscle power on aggravated glycemic control was higher in women than in men in this study. Furthermore, in women, there was a significant association between poor glycemic control and body composition, which was not found in men. A previous nationwide Korean survey showed a sex disparity in the risk factors for DM [37, 38]; abdominal obesity was associated with a 3 times higher risk of DM in women, which was not found in men [38]; this was consistent with a previous study carried out in a different population [39]. In contrast, the association between genetic factor and DM was more prominent in men than in women [38]. Although the reason for the differences in the association between body fat and DM between men and women is unknown, the prevalence of obesity increases in women who have a lower economic status; this was opposite in men [40]. Low economic status is a well-known risk factor for DM [40], which might partly explain the difference in the association between obesity and DM between the men and women. In addition, women have less muscle mass than men at any given age even after correction for height and weight [41]. This may explain more important role of muscle in metabolic health in women compared to that in men. Recently, a community-based cohort study also reported the deterioration of muscle function as well as reduction of muscle mass in diabetic patients was more prominent in women compared to men [42]; however, there have been conflicting data in the sex-difference in the association between sarcopenia and DM [43–45].

With aging, proliferation of β-cells reduce and apoptosis of β-cells increase; this gradually deteriorates pancreatic β-cell function [46]. However, in this study, there was no significant association between the primary and secondary outcomes and the surrogate markers of β-cell function. These findings might suggest that β-cell dysfunction has already been significantly reduced in older patients with long-standing diabetes, and insulin resistance worsens glycemic control than β-cell dysfunction. β-cell dysfunction is more common in patients with early-onset diabetes than in those with older age-onset diabetes [14]. In the older population, sarcopenia is the most significant factor for the development of glucose intolerance [14].

Our study had several limitations. First, the sample size was small. This may explain the absence of significant findings among the men after stratification: fewer men, when compared to women, had a low HGS. Second, HGS was not measured serially; therefore, it was not possible to determine the effect of the change of HGS on glycemic control. In addition, hyperglycemia itself results in impaired muscle function [31]; furthermore, improvement of glucose control increases muscle mass and function [47]. It might be difficult to exclude the effect of hyperglycemia at baseline on the association between muscle strength and hyperglycemia event during follow-up period. Last, genetic information that could help in excluding monogenic DM was unavailable; this may have affected the glycemic control [48]. However, using a prospective cohort of T2DM in a real-life clinical setting showed that muscle power and/or muscle mass played an important role in maintaining good glycemic control in older patients with long-standing T2DM.

## Conclusions

HGS is an important determinant in glycemic control in older patients with long-standing T2DM. Considering that an increase in muscle power and muscle mass can be achieved by resistance training in older patients even in the presence of CVD [49] or respiratory dysfunction [50], this study might provide a solid evidence for the importance of resistance training in older patients with T2DM.

## Supplementary Information

**Additional file 1: Table S1**. HbA1c level at the time point of each outcome. **Table S2**. Baseline clinical characteristics according to the secondary outcome. **Table S3**. Determinants of primary and secondary outcome in each sex. **Table S4**. Cox proportional hazards analysis for aggravation of glucose level according to low handgrip strength. **Fig. S1**. Cox proportional hazards model with adjustment for age, baseline HbA1c, obesity and duration of DM

## Data Availability

The datasets used and analysed during the current study are available from the corresponding author on reasonable request.
