# Supplementary material for: Muscle strength, an independent determinant of glycemic control in older adults with long-standing type 2 diabetes: a prospective cohort study
Source: BMC Geriatr. 2021 Dec 7;21:684. doi: 10.1186/s12877-021-02629-5 (PMC8650318; doi:10.1186/s12877-021-02629-5)

Supplemental Table 1. HbA1c level at the time point of each outcome

Event	N (%)	Median (interquartile range)
ΔHbA1c≥ 0.6%	38 (38.0)	7.85 (7.28–8.10)
ΔHbA1c< 0.6%	62 (62.0)	6.85 (6.5 –7.20)[a]
HbA1c≥ 9%	14 (14.0)	9.45 (9.18–10.08)
HbA1c< 9%	86 (86.0)	7.00 (6.60–7.43)[a]
Medication change due to hyperglycemia	71 (71.0)	8.00 (7.40–8.40)
No change in medication	29 (29.0)	7.10 (6.70–7.50)[a]

[a]HbA1c at the end of follow-up in the case without each outcome

Supplemental Table 2. Baseline clinical characteristics according to the secondary outcome

	Total (n = 100)	Secondary outcome		P^a
		No (n = 22)	Yes (n = 78)	
Age, year	63.9 ± 8.5	65.1 ± 8,1	63.6 ± 8.6	0.473
Men, n (%)	49 (49.0)	10 (45.5)	39 (50.0)	0.706
Duration of DM, years	20 (17 – 23)	19.5 (17 – 22)	20 (17 – 24)	0.782
BMI, kg/m^2	24.2 (22.1 – 26.6)	24.2 (22.0 – 26.5)	24.3 (22.0 – 26.9)	0.637
SBP, mmHg	129.0 ± 11.2	129.7 ± 11.5	128.9 ± 11.2	0.760
DBP, mmHg	75.8 ± 8.2	73.3 ± 8.7	76.5 ± 8.0	0.113
HbA1c, %	7.1 (6.7 – 7.4)	6.9 (6.6 – 7.7)	7.1 (6.7 – 7.4)	0.640
LDL cholesterol, mg/dL	79 (64 – 88)	84 (62 – 93)	78 (65 – 87)	0.451
HDL cholesterol, mg/dL	47 (40 – 54)	49 (44 – 58)	46 (39 – 53)	0.092
Triglyceride, mg/dL	103 (77 – 133)	84 (64 – 118)	104 (78 – 136)	0.220
AST	23 (18 – 30)	23 (19 – 29)	23 (18 – 30)	0.983
ALT	20 (15 – 28)	19 (15 – 27)	21 (16 – 28)	0.582
eGFR, mL/min/1.73m^2	85.4 (77.2 – 97.7)	84.9 (68.4 – 94.5)	85.6 (77.3 – 98.4)	0.326
eGFR ≥ 90, n (%)	38 (38.0)	7 (31.8)	31 (39.7)	0.747
eGFR 60–89, n (%)	56 (56.0)	14 (63.6)	42 (53.8)	
eGFR 30–59, n (%)	5 (5.0)	1 (4.5)	4 (5.1)	
ACR, mg/g	12.8 (7.5 – 26.2)	15.6 (9.8 – 41.7)	12.0 (7.1 – 25.5)	0.408
HOMA-IR	2.43 (1.95 – 3.86)	2.12 (1.66 – 3.04)	2.56 (2.02 – 4.01)	0.038
HOMA-β	44.2 (32.3 – 61.1)	44.7 (30.8 – 65.0)	44.2 (32.4 – 62.1)	0.947
IGI	1.21 (0.53 – 2.11)	1.54 (0.42 – 2.49)	1.20 (0.53 – 2.11)	0.780
c-peptide	2.26 (1.81 – 2.90)	2.14 (1.62 – 3.48)	2.37 (1.82 – 2.89)	0.589
Microalbuminuria, n (%)	21 (22.6)	6 (30.0)	15 (20.5)	0.562
Overt proteinuria, n (%)	1 (1.1)	0	1 (1.4)	
DMR, n (%)	40 (40.0)	8 (36.4)	32 (41.0)	0.136
NPDR, n (%)	27 (27.0)	6 (27.3)	21 (26.9)	0.237
PDR, n (%)	13 (13.0)	2 (9.1)	11 (14.1)	
Smoker, n (%)	28 (28.0)	4 (18.2)	24 (31.2)	0.233
Hypertension, n (%)	50 (50.0)	14 (63.6)	36 (46.2)	0.148
Obesity, n (%)	37 (37.0)	9 (40.9)	28 (36.4)	0.698
Statin, n (%)	58 (58.0)	13 (59.1)	45 (57.7)	0.907
Previous IHD, n (%)	5 (5.0)	0	5 (6.4)	0.407
Previous stoke, n (%)	4 (4.0)	2 (9.1)	2 (2.6)	0.168
Glucose-lowering agents				
Metformin	91 (91.0)	19 (86.4)	72 (92.3)	0.390
DPP4-inhibitors	64 (64.0)	12 (54.5)	52 (66.7)	0.296
SU	68 (68.0)	18 (81.8)	50 (64.1)	0.116
SGLT2 inhibitors	1 (1.0)	0	1 (1.3)	0.594
TZD	12 (12.0)	5 (22.7)	7 (9.0)	0.080

Secondary outcome was composed of ΔHbA1c≥ 0.6%, HbA1c≥ 9% and changing medication due to hyperglycemia. All values represent mean ± standard deviation or median (interquartile range) for

continuous variables and proportions (%) for categorical variables.
[a]Comparing the aggravation group and the maintenance group using Mann-Whitney test, independent t test and chi-square test

DM, diabetes mellitus; BMI, body mass index; SBP, systolic blood pressure; DBP, diastolic blood pressure; LDL, low-density lipoprotein; HDL, high-density lipoprotein; CRP, c-reactive protein; eGFR, estimated Glomerular filtration rate; ACR, albumin-to-creatinine ratio; NPDR, non-proliferative diabetic retinopathy; PDR, proliferative diabetic retinopathy; IHD, ischemic heart disease; DPP4, dipeptidyl peptidase 4; SU, sulfonylurea; SGT2, sodium glucose cotransporter; TZD, thiazolidinedione

Supplemental Table 3. Determinants of primary and secondary outcome in each sex

	Primary outcome		Secondary outcome	
	HR (95% CI)	*P*[a]	HR (95% CI)	*P*[a]
Male				
HOMA-IR≥2.5	2.155 (0.748, 6.205)	0.155	1.170 (0.579, 2.367)	0.662
HOMA-IR, per quartile	1.289 (0.868, 1.916)	0.208	1.146 (0.858, 1.552)	0.356
HOMA-IR, the highest quartile	1.398 (0.490, 3.983)	0.531	1.246 (0.593, 2.618)	0.562
HOMA-β, per quartile	1.048 (0.696, 1.576)	0.823	1.091 (0.788, 1.511)	0.598
HOMA-β, the lowest quartile	0.462 (0.142, 1.504)	0.200	0.874 (0.374, 2.046)	0.757
IGI, per quartile	1.168 (0.672, 2.031)	0.581	1.038 (0.710, 1.518)	0.847
IGI, the lowest quartile	0.781 (0.218, 2.790)	0.703	1.250 (0.475, 3.292)	0.651
C-peptide, per quartile	0.810 (0.469, 1.398)	0.449	1.163 (0.788, 1.716)	0.447
C-peptide, the lowest quartile	1.186 (0.271, 5.197)	0.821	0.497 (0.186, 1.326)	0.163
Muscle mass/BW, per quartile	1.790 (0.940, 3.408)	0.076	1.069 (0.712, 1.606)	0.747
Fat mass/BW, per quartile	0.670 (0.349, 1.285)	0.228	1.034 (0.636, 1.681)	0.893
Low handgrip strength	1.937 (0.399, 9.410)	0.412	0.666 (0.201, 2.203)	0.505
Age	1.040 (0.956, 1.131)	0.360	1.005 (0.964, 1.047)	0.826
BMI≥25 kg/m^2	1.612 (0.512, 5.075)	0.415	0.794 (0.387, 1.628)	0.528
Female				
HOMA-IR≥2.5	3.810 (1.307, 11.100)	0.014	2.264 (1.044, 4.910)	0.038
HOMA-IR, per quartile	1.669 (0.936, 2.978)	0.083	1.062 (0.705, 1.600)	0.772
HOMA-IR, the highest quartile	1.739 (0.320,9.444)	0.522	1.412 (0.505, 3.950)	0.511
HOMA-β, per quartile	1.255 (0.758, 2.077)	0.378	0.840 (0.573, 1.231)	0.371
HOMA-β, the lowest quartile	2.593 (0.817, 8.222)	0.106	1.634 (0.615, 4.342)	0.325

IGI, per quartile	0.690 (0.433, 1.098)	0.117	0.890 (0.647, 1.224)	0.473
IGI, the lowest quartile	2.722 (0.838, 8.843)	0.096	0.889 (0.393, 2.015)	0.779
C-peptide, per quartile	0.905 (0.551, 1.486)	0.694	1.071 (0.740, 1.551)	0.715
C-peptide, the lowest quartile	2.574 (0.704, 9.406)	0.153	1.863 (0.751, 4.617)	0.179
Muscle mass/BW, per quartile	0.458 (0.247, 0.850)	0.013	0.750 (0.480, 1.223)	0.249
Fat mass/BW, per quartile	1.970 (1.058, 3.667)	0.032	1.384 (0.853, 2.247)	0.188
Low handgrip strength	2.846 (1.090, 7.434)	0.033	2.298 (1.002, 5.374)	0.048
Age	0.986 (0.932, 1.042)	0.611	0.991 (0.948, 1.037)	0.704
BMI≥25 kg/m^2	0.328 (0.086, 1.255)	0.104	0.909 (0.396, 2.090)	0.823

Cox proportional hazards analysis for the primary and secondary outcome in each sex

[a] Adjusted for age, baseline HbA1c, obesity, duration of diabetes and anti-diabetic medication at baseline

Primary outcome was a composite of ΔHbA1c≥ 0.6% and HbA1c≥ 9%; secondary outcome was composed of ΔHbA1c≥ 0.6%, HbA1c≥ 9% and changing medication due to hyperglycemia.

Supplemental Table 4. Cox proportional hazards analysis for aggravation of glucose level according to low handgrip strength

	N =100	Unadjusted		Model 1		Model 2	
		HR (95% CI)	*P*	HR (95% CI)	*P*	HR (95% CI)	*P*
Primary outcome	40 (40.0)	2.274 (1.132, 4.567)	0.021	2.543 (1.231, 5.252)	0.012	2.234 (1.058, 4.718)	0.035
Secondary outcome	78 (78.0)	1.498 (0.848, 2.648)	0.164	1.720 (0.942, 3.141)	0.077	1.556 (0.838, 2.887)	0.161
Each composite outcome							
ΔHbA1c≥ 0.6%	38 (38.0)	2.547 (1.258, 5.155)	0.009	2.793 (1.334, 5.847)	0.006	2.467 (1.149, 5.300)	0.021
HbA1c≥ 9%	14 (14.0)	2.534 (0.848, 7.569)	0.096	3.313 (1.059, 10.361)	0.040	2.490 (0.715, 8.678)	0.152
Medication change	71 (71.0)	1.346 (0.749, 2.420)	0.321	1.707 (0.912, 3.193)	0.094	1.526 (0.796, 2.928)	0.203

Cox proportional hazards analysis for the primary, secondary and each composite outcome according to low hand grip strength
Model 1: adjusted for age, sex, and baseline HbA1c
Model 2: adjusted for obesity, duration of diabetes and anti-diabetic medication at baseline in addition to Model 1
Primary outcome was a composite of ΔHbA1c≥ 0.6% and HbA1c≥ 9%; secondary outcome was composed of ΔHbA1c≥ 0.6%, HbA1c≥ 9% and changing medication due to hyperglycemia.

Supplementary Figure 1

Cox proportional hazard model with adjustment for age, baseline HbA1c, obesity, duration of DM and anti-diabetic medications at baseline

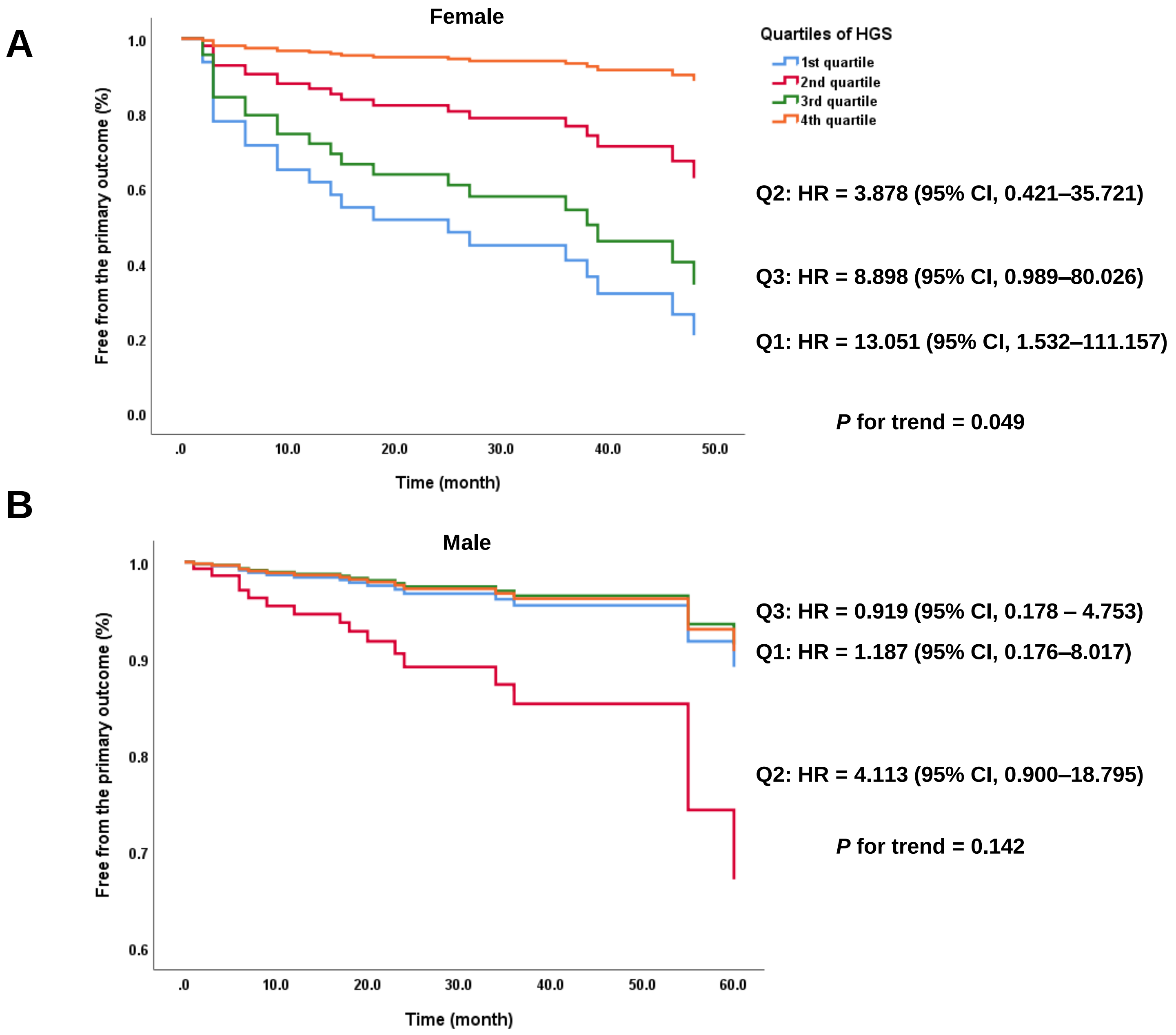

Supplement: Supplementary file 1 — Additional file 1: Table S1. HbA1c level at the time point of each outcome. Table S2. Baseline clinical characteristics according to the secondary outcome. Table S3. Determinants of primary and secondary outcome in each sex. Table S4. Cox proportional hazards analysis for aggravation of glucose level according to low handgrip strength. Fig. S1. Cox proportional hazards model with adjustment for age, baseline HbA1c, obesity and duration of DM [file 12877_2021_2629_MOESM1_ESM.pdf]
